# Biological Therapy in Systemic Lupus Erythematosus

**DOI:** 10.1155/2012/578641

**Published:** 2012-01-30

**Authors:** Mariana Postal, Lilian TL Costallat, Simone Appenzeller

**Affiliations:** Rheumatology Unit, Department of Medicine, Faculty of Medical Science, State University of Campinas, 13083-887 Campinas, SP, Brazil

## Abstract

Systemic lupus erythematosus (SLE) is a prototypic inflammatory autoimmune disorder characterized by multisystem involvement and fluctuating disease activity. Symptoms range from rather mild manifestations such as rash or arthritis to life-threatening end-organ manifestations. Despite new and improved therapy having positively impacted the prognosis of SLE, a subgroup of patients do not respond to conventional therapy. Moreover, the risk of fatal outcomes and the damaging side effects of immunosuppressive therapies in SLE call for an improvement in the current therapeutic management. New therapeutic approaches are focused on B-cell targets, T-cell downregulation and costimulatory blockade, cytokine inhibition, and the modulation of complement. Several biological agents have been developed, but this encouraging news is associated with several disappointments in trials and provide a timely moment to reflect on biologic therapy in SLE.

## 1. Introduction

Systemic lupus erythematosus (SLE) is an autoimmune, multisystemic, relapsing, and remitting disease that is characterized by the production of antibodies against nuclear antigens. The pathogenesis includes genetic, environmental, and hormonal factors, but the cause of SLE remains unclear. A broad array of clinical manifestations ranging from mucocutaneous and arthritis to severe organ- and life-threatening disease are observed in SLE patients [1, 2].

The current treatment options include the use of corticosteroids, hydroxychloroquine, and other immunosuppressive medications (e.g., azathioprine, mycophenolate, and cyclophosphamide) [2]. More recently, belimumab was approved by the FDA for SLE treatment [3].

Due to earlier diagnosis and better treatment options of both disease and complications, the prognosis has markedly improved in the last decades. The 5-year survival of patients with SLE has exceeded 90% in most centers [4, 5]. However, morbidity, especially renal failure, and mortality from cardiovascular events after long-term followup are still an important issue [5].

In the last decade new treatment strategies have been developed. Advanced knowledge of the pathogenesis of SLE has led to new therapeutic approaches targeting specific molecules [4]. Beside autoantibody production, B-cells are the key for the activation of the immune system, particularly through cytokines and as antigen-presenting cells. An important part of B-cells is activated in a T-cell-dependant manner.

This paper will review the rational of biologic therapies in SLE and discuss potential therapeutic options.

## 2. B-Cell Targets

B cells have been largely implicated in the pathogenesis of SLE as sources of autoantibody, as antigen-presenting cells, and as initiators and regulators of inflammation through cytokine secretion [6–8]. B-cell-targeted therapies, including anti-CD20 monoclonal antibody (Rituximab) and anti-B lymphocyte stimulator (BLyS), are at forefront of new SLE therapies [8, 9] (Table 1).

### 2.1. Anti-CD20 Antibody

**Figure 1:**
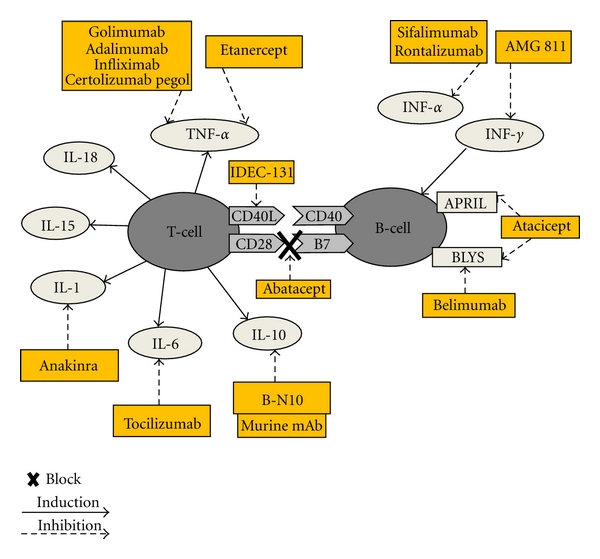
Potential targets and relevant drugs in connection with B and T cells in the management of SLE.

The first B-cell depleting antibody used in SLE was rituximab, a chimeric murine/human monoclonal antibody against CD20 (Figure 1). CD20 is expressed early in the development of B lymphocytes. Rituximab administration results in rapid depletion of CD20-positive B lymphocytes [7, 38, 39]. After rituximab treatment some patients reconstitute with naive B cells and enter remission. Others, however, do not deplete B cells completely and they reconstitute with memory B cells and might therefore benefit from rituximab retreatment [10]. Two recent open-label studies confirmed that repeated cycles of rituximab are effective in treating refractory SLE, may produce a sustained clinical response and have a favorable safety profile [10, 11].

Rituximab has been used in open trials and improvements in disease activity has been observed [12, 13]. In addition it has been shown to be safe and well tolerated [13, 40, 41]. Two large multicenter randomized placebo-controlled trials with rituximab in moderately to severely active SLE (EXPLORER) [14] and in proliferative lupus nephritis patients (LUNAR) [15] could not demonstrate a significant benefit of rituximab when compared to placebo. The inclusion of milder forms of SLE, the ethnic background of patients, the concomitant use of steroids and other immunosuppressive drugs, and the short followup (52 weeks) could explain in part why no benefit could be demonstrated for rituximab in these studies [14, 15, 42]. Despite the lack of evidence in randomized trials, rituximab has been used in refractory patients and improvement in up to 89% of the patients has been observed [43–47].

 Adverse events associated with the use of rituximab are most often mild, but infusion reactions (30–35%), neutropenia (8%), and human antichimeric antibodies (9%) production have been observed [10]. In addition, two cases of fatal progressive multifocal leukoencephalopathy (lethal encephalitis caused by the polyomavirus JC) in SLE patients after rituximab treatment have been reported [11].

 New monoclonal anti-CD20 antibodies have been developed. Ocrelizumab, a recombinant humanized monoclonal anti-CD20 antibody has been studied in Phase III trials in extrarenal SLE (BEGIN study) [16] and lupus nephritis (BELONG study) [17]. However, treatment with ocrelizumab has been suspended in SLE trials, following the negative outcome of a similar study design with the anti-CD20 antibody and also due to an increase in the treatment group [16, 17, 48].

### 2.2. Anti-CD22 Antibodies

Epratuzumab is a fully humanized antibody against CD22. CD22 is 128a 135-kD B-lymphocyte restricted type I transmembrane sialoglycoprotein of the Ig superfamily and modulates B-cell function without B-cell depletion [9, 49]. Epratuzumab was evaluated in randomized controlled trials in patients with moderate-to-severe SLE flares [18]. An improvement in BILAG scores and reduction in corticosteroid doses with a good safety profile was observed; however the trial was interrupted due to problems in the biologic supply [18]. Two studies are currently evaluating the efficacy of epratuzumab in a subset of serologically active SLE, and results have yet not been presented [19, 20].

### 2.3. B-Lymphocyte Tolerogens

Abetimus (LJP-394) is a B-cell tolerogen. It consists of four double-stranded DNA (dsDNA) epitopes on a polyethylene glycol platform [50]. It cross-links anti-dsDNA surface immunoglobulin receptors on B-cells, leading to anergy or apoptosis. It also reduces titers of anti-dsDNA antibodies [21]. Abetimus was the first B-cell tolerogen developed for SLE and was studied in human trials for the treatment of nonrenal lupus and lupus nephritis [21]. Initial trials suggested a reduction in renal flares in patients who have high-affinity antibodies to the DNA epitope contained within the abetimus molecule [4, 21]. After an analysis of a phase III Abetimus Sodium in patients with a history of lupus nephritis (ASPEN) trial, the trial was terminated when interim efficacy analysis indicated no benefit to continue [51].

Another tolerogen, TV-4710 (Edratide) a peptide composed of 19 amino acids based on the complementarily determining regions (CDR1) of a human anti-dsDNA antibody, was tested in a phase II trial [22]. This study has been concluded but there are yet no results released [22].

### 2.4. BLyS Blockers

The B-cell survival molecule B-lymphocyte stimulator (BLyS) also known as B-cell activation factor of the TNF family (BAFF) plays a key role in the activation and differentiation of B cells [4]. BLyS represents, therefore, an excellent target for interventions in SLE. High serum levels of soluble BLyS, and its homolog APRIL (a proliferation inducing ligand), are found in SLE patients and in murine lupus. Selective blockade of BLyS reduces transitional type 2 follicular and marginal-zone B cells and significantly attenuates immune activation [4, 9].

Belimumab is a fully human monoclonal antibody that binds to BLyS and inhibits its biological activity (Figure 1). Efficacy, tolerability, and safety of three different doses of belimumab in SLE were evaluated in a multicenter phase II study [23]. After 52 weeks of analysis, belimumab was associated with a reduction in activity and new flares. Two phase III trials (BLISS-52 and BLISS-76) showed that belimumab plus standard care achieved a significant improvement in patient response rate and increased time to-first-flare compared with placebo plus standard care [23, 52]. Based on these results, FDA recently approved Belimumab for the treatment of SLE [3].

 An alternative blocker to BlyS is atacicept (also known as TACI-Ig). It is a soluble transmembrane activator and calcium-modulator and cyclophilin ligand interactor (TACI) receptor, which binds both BAFF and APRIL (Figure 1). In a phase I trial in SLE patients, atacicept was well tolerated [24].

 Atacicept is of interest in SLE because of its profound effects on plasma cells, but its use leads to significant decrease in IgM and IgG immunoglobulin levels [53, 54]. A phase II study of atacicept plus mycophenolate in SLE nephritis was terminated because of an increased number of infections [53]. The increased number of infection could be explained by the fact that plasma cells require APRIL and so serum Ig was reduced. A phase II/III trial of atacicept for generalized SLE (April SLE) is still ongoing [55].

## 3. T-Cell Target and Costimulatory Blockers

Costimulatory molecules provide the necessary second signal for T-cell activation by antigen-presenting cells. The inhibition of this mechanism has been demonstrated to be effective in murine lupus models [56, 57]. The most important antigen-independent signal for T-cell activation is the CD28:B7 costimulatory interaction [4]. CD28 is expressed on T cells, whereas the ligands B7-1 and B7-2 (CD80 and CD86) are found on antigen-presenting cells [4]. CTLA4 inhibits T-cell activation by binding to B7-1 and B7-2 (CD80 and CD86) expressed on antigen-presenting cells. Therefore CTLA4 interacts with B7 but inhibits T-cell activation, by preventing the costimulatory signal CD28-B7 interaction necessary for T-cell activation [4] (Figure 1).

 Abatacept is a soluble receptor or fusion protein encoded by fusion of CTLA-4 with the Fc portion of IgG1. Abatacept blocks CD28-B7 interaction and subsequent T-cell-dependent B-cell function [58, 59] (Figure 1). In murine model, abatacept prevents initiation but not evolution of antiphospholipid syndrome in NZW/BXSB mice [59]. In SLE patients, abatacept has been tested in phase I to III trials [25, 26].

 CD40-CD40 ligand (CD40L) is another important costimulatory pair that induces T-cell-dependent B-cell proliferation and antibody production. CD40 is expressed on B cells, endothelial cells, and antigen-presenting cells and binds to CD40L (or CD154) on CD4+ T helper cells [4] (Figure 1). In lupusprone mice with nephritis treated with anti-CD40L antibodies reduction in anti-dsDNA antibody, milder renal disease and increased survival was observed [60]. Unfortunately, anti-CD40L monoclonal antibody (mAb) (IDEC-131) did not prove to be clinically effective in human SLE compared with placebo [27]. Another study (BG9588) was terminated prematurely after a few patients demonstrated life-threatening prothrombotic events despite improvement in serologic activity [61].

 Efalizumab is a monoclonal antibody directed against CD11a, the alpha-subunit of the leukocyte-functioning antigen-1. It plays an important role in T-cell activation, re-activation, extravasation, and trafficking from the circulation into the skin, through its binding to intercellular adhesion molecules (Figure 1). Efalizumab seems to reduce cutaneous manifestations in SLE patients [28]. The majority of patients with difficult lupus discoid had an important response to treatment with the mean time to response being 5.5 week [28]. However, this study evaluated only a small number of patients. There is a need for more prospective studies with long-term followup to better define the efficacy and safety of efalizumab in SLE.

The inducible costimulator (ICOS) is a T-cell-specific molecule structurally and functionally related to CD28. ICOS regulates T-cell activation and T-helper cell differentiation and is mainly involved in humoral immune responses and, thus, autoantibody production. A fully humanized anti-B7RP1 antibody (AMG557) is currently being investigated and may represent a further target for SLE therapy [29].

Mammalian target of rapamycin (mTOR) has multiple regulatory functions in T- and B-cell intracellular signaling [62]. It controls the expression of T-cell receptor-associated signaling proteins through increased expression of the endosome recycling regulator genes and enhances intracellular calcium flux [63]. Rapamycin (Sirolimus) interacts with mTOR by influencing gene transcription and multiple cellular metabolic pathways. This interaction has been proven to be beneficial in murine lupus [64]. Rapamycin appeared to be a safe and effective therapy for refractory SLE in a small pilot study [30].

## 4. Cytokine Inhibition

As cytokine dysregulation can be demonstrated in murine and in SLE patients, an anticytokine approach seems promising in this autoimmune disease [65]. Cytokines such as tumor necrosis factor alpha (TNF-*α*), interferon alpha and gamma (IFN-*α*/-*γ*) and interleukins (IL) 1, 6, 10, 15, and 18 are upregulated in SLE and play important roles in the inflammatory processes that leads to tissue and organ damage [65]. These cytokines have been considered potential targets for the reduction of chronic inflammation in SLE (Figure 1).

### 4.1. Anti-TNF-*α*

TNF-*α* is a pleiotropic cytokine that exerts several functions in the immune system and can either promote or reduce autoimmunity. In SLE, its role is controversial. TNF-*α* promotes apoptosis and significantly affects the activity of B and T cells and dendritic cells (DCs). In different strains of lupus mice, the expression of TNF-*α* is often variable, and beneficial effects on the disease can be observed either after administration of TNF-*α* or upon TNF-*α* blockade [31, 66–68]. TNF-*α* blockers are associated with the development of autoantibodies, such as antinuclear, anti-dsDNA, and anticardiolipin, as well as with rare cases of drug-induced lupuslike syndromes, all of which disappear after therapy is discontinued [65].

 There are several TNF-*α* inhibitors available for clinical use such as infliximab, adalimumab, golimumab, and certolizumab pegol and a fusion protein that acts as a “decoy receptor” for TNF-*α* (etanercept) [31, 69] (Figure 1). TNF-*α* inhibitors are usually well tolerated; however their use may increase the overall risk of opportunistic infections, in particular the reactivation of latent tuberculosis [70, 71]. The appearance of neutralizing antibodies has been described in patients treated with infliximab, which is a chimeric human/mouse mAb, as well as in those treated with adalimumab, in spite of its fully human sequence [71]. The concomitant use of an immunosuppressive drug like methotrexate has been shown to prevent the development of neutralizing antibodies [72].

### 4.2. Anti-IFN-*α*/-*γ*

IFN-*α* plays a significant role in the pathogenesis of SLE. IFN-*γ* is elevated in (New Zealand Black [NZB] × New Zealand White [NZW]) F1 (NZB/W) lupus mice, and a correlation with disease activity has been observed [73, 74]. In addition, administration of IFN-*γ* accelerates murine lupus, while anti-IFN-*γ* antibody (or soluble IFN-*γ* receptor or IFN-*γ* receptor-immunoglobin) delays the disease [75–77]. Finally, it has been demonstrated that late treatment with IFN-*γ* in MRL/lpr mice accelerates SLE, while early treatment protects disease progression [78]. IFN-*α* levels are increased in SLE patients and correlate with disease activity and kidney involvement [79]. In addition an increased expression of interferon-regulated inflammatory genes in the peripheral blood mononuclear cells of the SLE patients (known as “interferon signature”) has been observed [80, 81].

 Sifalimumab (MEDI-545) is a monoclonal human antibody that blocks multiple IFN-*α* subtypes. It is currently being tested in phase I/II clinical trials to evaluate safety and tolerability of multiple intravenous and subcutaneous doses in SLE [32] (Figure 1). 

Rontalizumab, a humanized mAb against IFN-*α* (rhuMAb IFN-*α*) is in a phase II, randomized, double-blind, placebo-controlled trial that evaluates the efficacy and safety in patients with moderately to severely active SLE [33] (Figure 1).

AMG 811, a human mAb to IFN-*γ*, is under investigation in a phase Ib, randomized, multicenter study in SLE patients with and without glomerulonephritis [34].

### 4.3. Anti-IL-1

IL-1 levels are increased by serum TNF levels and by anti-dsDNA antibody. The increase in serum IL-1 level is associated with lupus disease activity and a low level of IL-1 receptor antagonist is seen in patients with lupus nephritis [82, 83]. Anakinra, a nonglycolysated version of the human IL-1Ra (IL-1 receptor antagonist), neutralizes the biological activity of IL-1 (Figure 1). It has been used as an alternative in individual patients with lupus arthritis not responding to conventional treatments [35]. Anakinra has shown both safety and efficacy in improving arthritis in an open trial on four SLE patients, however short-lasting therapeutic effects were observed in two patients [35].

### 4.4. Anti-IL-6

IL-6 induces B-cell differentiation to plasma cells, hyperactivity, and secretion of antibodies and also promotes T-cell proliferation, cytotoxic T-cell differentiation, and local inflammation [65]. IL-6 is highly expressed in patients with lupus nephritis. IL-6 is induced in DCs by nucleic acid containing immune complexes as well as by multiple cytokines, including TNF, IL-1, and IFN-*γ*. In NZB/W mice IL-6 promotes disease, and anti-IL-6 therapy delays lupus nephritis, suggesting that IL-6 blockade might also be beneficial in SLE patients [84].

Tocilizumab is a humanized IgG1 antibody directed to human IL-6 receptor that inhibits IL-6 signaling [85] (Figure 1). An open-label, dose escalating phase I study of tocilizumab in SLE patients has recently been published [36]. Although neutropenia may limit the maximum dosage of tocilizumab in SLE patients, the observed clinical and serologic responses are promising and warrant further studies to establish the optimal dosing regimen and efficacy [36].

### 4.5. Anti-IL-10

IL-10 is produced by Th2 cells and considered an inhibitory cytokine for T cells and contrasts the activity of other proinflammatory cytokines such as TNF-*α* and IFN-*γ*. In SLE patients, IL-10 levels are increased in sera and are associated with disease activity [50]. NZB/W mice treated with anti-IL-10 mAb have reduced anti-dsDNA antibody titers and a delay in the onset of proteinuria and glomerulonephritis [86].

In the absence of a humanized mAb to IL-10, the murine anti-IL-10 mAb (B-N10) was used to inhibit the activity of IL-10 in a small uncontrolled, open-label study in SLE patients with relatively mild disease [37] (Figure 1). Disease activity improved and inactivity was observed in SLE patients up to 6 months after treatment. However, all patients developed antibodies against the murine mAb [37].

### 4.6. Anti-IL-15

IL-15 is mainly produced by the macrophage/monocyte cell line [87]. High serum levels of IL-15 are found in 40% of SLE patients; however its levels are not directly associated with disease activity [87]. IL-15 might be responsible for some immune abnormalities of the disease, such as stimulating lymphocytic expression of B-cell lymphoma 2 (Bcl-2) and CD25 (in both B and T-cells) [87]. Therapeutic agents against IL-15 are currently being tested in other autoimmune diseases.

### 4.7. Anti-IL-18

IL-18 is a proinflammatory cytokine closely related to IL-1. Several groups have observed increased serum levels of IL-18 in SLE patients, which appear to be associated with TNF levels [88–90]. IL-18 is overexpressed in the nephritic kidneys of MRL/lpr mice. Moreover, MRL/lpr mice benefit from targeting IL-18 [91]. Until now, IL-18 blockade has not been used in human SLE (Figure 1).

## 5. Complement Inhibition

The complement system consists of 3 pathways and more than 30 proteins, including those with biological activity that directly or indirectly mediate complement effects, plus a set of regulatory proteins necessary to prevent inadvisable complement activation [9]. The complement system appears to have a protective effect in SLE, since homozygous deficiencies of classic pathway components are associated with an increased risk for SLE. The deposition of immune complexes, however, observed in human and animal models, leads to an activation of the complement system, amplifying the inflammatory response. Pathologic evidence of immune complex-mediated activation of complement in affected tissues is clearly evident in both experimental and human SLE [92].

 Two complement inhibitors, soluble complement receptor 1 (TP10) and a monoclonal anti-C5 antibody (Eculizumab) have been shown to inhibit complement safely and now are being investigated in a variety of clinical conditions [9]. Eculizumab has shown to reduce hemolysis and has been approved by the FDA in paroxysmal nocturnal hemoglobinuria [93]. Although still no clinical trial has been performed in SLE, they hold promise to be used therapeutically in SLE [93].

## 6. Conclusion

In recent years advances in our understanding of the mechanisms of SLE has offered better drug targets for treatment. Over the next years, we will test the efficacy of many new therapeutic agents. The knowledge on how to divide patients into subsets according to genetic susceptibility, pathogenetic mechanisms, and phases of the disease will maximize the therapeutic effect of each agent and minimize its toxicity.

## Figures and Tables

**Table 1:** Biological therapies proposed for SLE treatment.

Biologic drug	Main results
B-cell targets	

Anti-CD20 antibody	
Rituximab	Effective in treating refractory SLE [10, 11] Improvements in disease activity [12, 13] No benefit in proliferative lupus nephritis [14, 15]
Ocrelizumab	No benefit in lupus nephritis [16, 17]
Anti-CD22 antibody	
Epratuzumab	Improvement in BILAG scores [18] Reduction in corticosteroid doses with a good safety profile [19, 20]
B-lymphocyte tolerogens	
Abetimus	No long-term benefit in patients with lupus nephritis [21]
Edratide	No results released [22]
BLyS blockers	
Belimumab	Reduction in activity and new flares [23]
Atacicept	Significant decrease in IgM and IgG levels [24]

T-cell target and costimulatory blockers	

Abatacept	Improvements in non-life- threatening SLE manifestations [25, 26]
IDEC-131	No clinically effective in human SLE [27]
Efalizumab	Reduction in cutaneous SLE manifestations [28]
AMG557	No results released [29]
Sirolimus	Safe and effective for refractory SLE [30]

Cytokine inhibition	

Anti-TNF-*α*	
Infliximab	Long-term efficacy for lupus nephritis [31]
Anti-IFN-*α*/-*γ*	
Sifalimumab Rontalizumab AMG 811	No results released [32] No results released [33] No results released [34]
Anti-IL-1	
Anakinra	Improvements in SLE arthritis [35]
Anti-IL-6	
Tocilizumab	Improvements in clinical and serologic responses [36]
Anti-IL-10	
B-N10^a^	Improvements in disease activity [37]

^a^Murine Lupus; BILAG: The British Isles Lupus Assessment Group; BLyS: B cell survival molecule B lymphocyte stimulator; Ig: immunoglobulin; TNF: tumor necrosis factor; INF: interferon; IL:interleukin.

## Abbreviations

TNF-*α*: Tumor necrosis factor alpha
IFN-*α*/*γ*: Interferon alpha and interferon gamma
IL: Interleukin
mAb: Monocloral antibodies
BLyS: B lymphocyte stimulator
APRIL: Proliferation inducing ligand
CTLA-4: Cytotoxic T lymphocyte-associated antigen 4.
